# Methylation of Cyanidin-3-*O*-Glucoside with Dimethyl Carbonate

**DOI:** 10.3390/molecules26051342

**Published:** 2021-03-03

**Authors:** Sarah Straßmann, Tillman Brehmer, Maike Passon, Andreas Schieber

**Affiliations:** Department of Nutritional and Food Sciences, Molecular Food Technology, University of Bonn, Friedrich-Hirzebruch-Allee 7, D-53115 Bonn, Germany; sarah.strassmann@magenta.de (S.S.); brehmer@uni-bonn.de (T.B.); schieber@uni-bonn.de (A.S.)

**Keywords:** cyanidin-3-*O*-glucoside, cyanidin, anthocyanins, LC-IMS, HRMS, methylation, DMC, phase II metabolites

## Abstract

The approach presented in this study is the first for the hemisynthesis of methylated anthocyanins. It was possible to obtain cyanidin-3-*O*-glucoside derivatives with different degrees of methylation. Cautious identification of 4′-, 5-, and 7-OH monomethylated derivatives was also accomplished. The methylation agent used was the “green chemical” dimethyl carbonate (DMC), which is characterized by low human and ecological toxicity. The influence of the temperature, reaction time, and amount of the required diazabicyclo[5.4.0]undec-7-en (DBU) catalyst on the formation of the products was examined. Compared to conventional synthesis methods for methylated flavonoids using DMC and DBU, the conditions identified in this study result in a reduction of reaction time, and an important side reaction, so-called carboxymethylation, was minimized by using higher amounts of catalyst.

## 1. Introduction

Anthocyanins are natural plant colorants and are a component of fruit and vegetables in the human diet [1]. Like other polyphenols, they show antioxidant properties, e.g., against radical oxygen species. As such, they are associated with the prevention of certain diet-related illnesses, such as cardiovascular disease and cancer [2]. The increased interest in anthocyanin-rich nutrition is accompanied by the elucidation of the physiological relationships between intake and metabolism. Metabolites of anthocyanins are inter alia compounds glucuronidated, sulfated, and/or methylated at the aglycone [3]. Knowledge of the structure and quantity of these metabolites after consumption of anthocyanin-rich meals in the human body provides information about the intake and bioavailability and thus, in the long term, about the health significance of anthocyanins in vivo [4]. For such investigations, standard substances are required, and their production gives rise to new research areas [5].

In living organisms, conjugation of flavonoids having a catechol structure with methyl groups is carried out by catechol-*O*-methyl transferase enzymes (COMT) and *S*-adenosylmethionine (SAM) as a coenzyme [6]. Since cyanidin has this catechol structure only on the B ring, there is no methylation of the A ring. Consequently, methylated derivatives observed so far in vivo carry the methyl group either at the 3′-(peonidin-3-*O*-glucoside) or at the 4′-hydroxyl group of cyanidin-3-*O*-glucoside (isopeonidin-3-*O*-glucoside) [7]. The metabolites themselves can either be excreted directly or be subject to further metabolism. The physiological properties of the metabolites may differ from those of the native starting compounds [8].

Metabolically significant methylated reference substances can be synthesized using different approaches. A promising approach for glucuronidation, sulfation, and methylation is enzymatic hemisynthesis, but costs and efforts are too high to result in usable quantities [9]. Due to the enzyme properties, another drawback of this synthesis route is the lack of the ability to generate derivatives with more than one methyl group, which can be relevant for already genuinely methylated compounds.

Thus, chemical synthesis is more promising. This may either mean full synthesis, as performed successfully by Cruz et al. [10], or hemisynthesis. The advantage of hemisynthesis is that only a few steps are required to achieve the goal. Initially, a suitable methylation agent is required. For flavonoids such as quercetin or catechin, successful syntheses with dimethyl sulfate (DMS) or methyl iodide, also known as iodomethane, have been described [5,11]. In addition, mono-, di- and tetramethylated naringenin derivatives were synthesized by using anhydrous acetone and *N,N*-dimethylformamide (DMF), respectively [12]. Since DMS and methyl iodide are toxic and carcinogenic, working with dimethyl carbonate (DMC) is an attractive alternative.

**Scheme 1:**
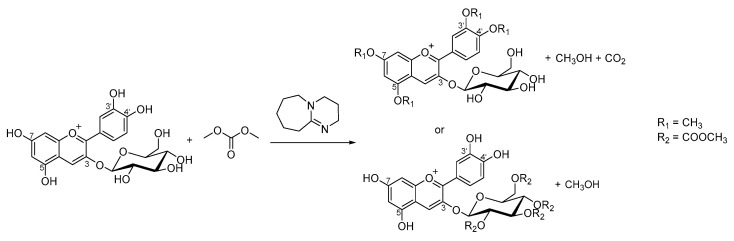
Possible reactions of cyanidin-3-*O*-glucoside with DMC and DBU.

For DMC, two reactions are of particular importance: carboxymethylation, also known as methoxycarbonylation, at low temperatures, and methylation at temperatures above 120 °C [13]. Both reactions are shown in Scheme 1.

The crucial difference in carboxymethylation and methylation lies in the mechanism of base-mediated ester hydrolysis of DMC. Successful *O*-methylation (in the following referred to as methylation) depends on a suitable catalyst to prevent carboxymethylation [14]. Methylation and carboxymethylation are also dependent on the type of alcohol [15]. Thus, methylation is preferred on aryl alcohols and carboxymethylation on aliphatic alcohols. Most syntheses of phenols with DMC take place at temperatures above its boiling point, i.e., between 120 and 200 °C [16,17]. Considering the sensitivity of anthocyanins to high temperatures and long reaction times [18], this is rather critical for their methylation. By using diazabicyclo[5.4.0]undec-7-ene (DBU) as a catalyst, the temperature of the methylation can be reduced below 100 °C, [19] which is nevertheless still too high for the direct synthesis of the cyanidin aglycone.

The synthesis of methylated anthocyanins for the exploration of metabolites in physiological samples poses a special challenge, as only aglycone-substituted compounds are relevant. Although the aglycone is an aryl alcohol, the glucose moiety is an aliphatic alcohol. These types differ in the way they react with DMC. Thus, the aim of the synthesis was to determine the reaction conditions for highly selective *O*-methylation of the aglycone without carboxymethylation of the sugar. For cyanidin-3-*O*-glucoside, these are the derivatives methylated at the B ring [7]. Furthermore, the yield of mono- and dimethylated derivatives should be maximized as much as possible, as they are interesting from a metabolic point of view. At the same time, the amount of higher methylated compounds should be minimized.

## 2. Results and Discussion

Using the methods described above, three monomethylated, three dimethylated, three trimethylated, and one tetramethylated derivative were generated (Table 1). In addition, three carboxymethylated compounds were detected. The path that led to these results and the identification of the monomethylated derivatives is described below.

### 2.1. First Observations

Simple hemisyntheses of monomethylated quercetin derivatives with DMS take between 4 and 24 h, depending on the solvents and bases used [20]. The synthesis of monomethylated flavonoids with DMC requires 24 to 36 h according to the literature [21]. In the first experiments with 10 mg of anthocyanin extract, 2 mL DMC, and 0.3 mmol DBU at 90 °C, the compounds that were fully methylated could already be identified as the main products after 22 h. The samples contained methylated cyanidin-3-*O*-glucoside at a mass to charge ratio (*m*/*z*) 505 and 563 ([M]^+^). The former is the derivative that is fully methylated at the aglycone, whereas the latter is carboxymethylated in addition to permethylation. No monomethylated derivatives were detectable in the samples examined. For this reason, it was decided to shorten the reaction time to stop the reaction at the point where compounds with a lower degree of methylation, such as monomethylated molecules at *m*/*z* 463 ([M]^+^) and dimethylated molecules at *m*/*z* 477 ([M]^+^) are formed. The optimal reaction time, temperature, and quantity of the catalyst for the formation of noncarboxymethylated monomethylated cyanidin-3-*O*-glucoside derivatives can be determined with the help of a randomized, central composite design.

### 2.2. Experimental Design

In the synthesis presented here, it is presumed that the nonnucleophilic base, DBU, deprotonates cyanidin-3-*O*-glucoside. In addition, DBU reacts with DMC via the preferred carboxymethylation mechanism to generate a more activated methylating agent. This reacts in the next step with the deprotonated cyanidin-3-*O*-glucoside to form the desired methylated compound while the catalyst is regenerated. The resulting methoxycarbonate anion decays rapidly to CO_2_, and, with a proton, to methanol. It becomes clear that the DBU added is only a catalyst. The mechanism was previously described [13,19], but still needs to be elucidated in more detail. Since anthocyanins have a pH-dependent structure, this certainly plays a major role in the reaction. It is expected that the flavylium cation form, which is present in acidic environments, is more stable and less reactive than the deprotonated form.

**Figure 1:**
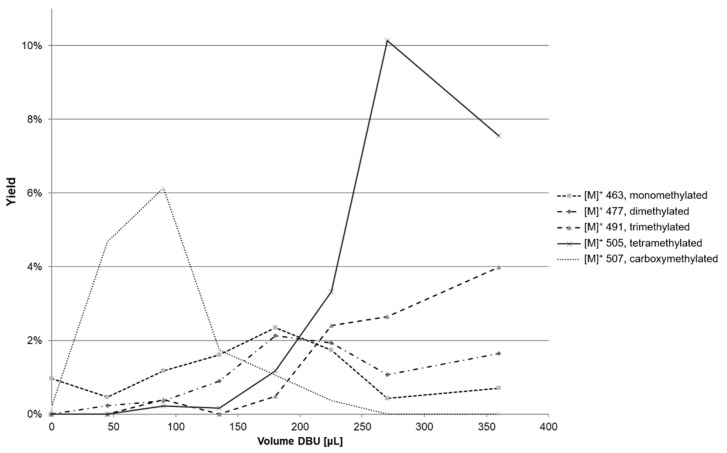
Yield of the synthesis products of cyanidin-3-*O*-glucoside with 2 mL DMC at 90 °C for 6 h as a function of the volume of catalyst DBU.

Figure 1 shows the relationship between the yield of synthesized compounds at 90 °C for six hours and the volume of catalyst.

The tri- and tetramethylated compounds are formed only at higher amounts of DBU, while the amount of mono- and dimethylated derivatives decreases at this point. It should be mentioned that small amounts of peonidin-3-*O*-glucoside were sometimes detected in the blackberry extract used as the starting material. A consideration of the undesired side reaction of carboxymethylation shows that these reactions can be controlled by the concentration of DBU. Without catalyst, the product at *m*/*z* 507 ([M]^+^) is not formed, but with 0.6 mmol DBU, its yield is over 6%. This small amount of DBU is immediately consumed as a base and cannot be used to generate the methylation agent. In this way, the anthocyanin reacts directly with DMC, so carboxymethylation is preferred. This consumption of DBU is also described in the literature [19]. With increasing catalyst quantities, a decrease in carboxymethylated cyanidin-3-*O*-glucoside can be observed. With more than 1.2 mmol DBU, the concentration of carboxymethylated cyanidin-3-*O*-glucoside is no longer significant. The result is plausible since DBU reacts as a base and creates the methylating agent from DMC, and thus, only the methyl group is transferred to the cyanidin-3-*O*-glucoside. This pathway also produces CO_2_ as the driving force of the reaction.

These assumptions could be confirmed in tests with different concentrations of the starting compounds but the same amount of catalyst. Increasing the amount of methylating agent only promotes the side reaction of carboxymethylation. In one of the reactions investigated, a yield greater than 8% was achieved. From the synthesis of *p*-cresol to 4-methyl anisole described in the literature, it can be concluded that a substrate concentration that is too high slows down the reaction kinetics. The reason for this effect is the possibility of the substrate interfering with the formation of the phenolate ion, which is necessary for the transfer of the methyl group, by forming hydrogen bonds between them [22]. A similar connection is also likely for anthocyanins. With 1 mL of DMC but the same anthocyanin-to-catalyst ratio, the yield decreases compared to the optimized conditions with 2 mL. Thus, it may be concluded that in case of an excess of methylating agent and a too low concentration of the catalyst, the direct contact of DMC with the anthocyanin leads to carboxymethylation of the sugar residue.

The experimental design also confirmed the assumption that carboxymethylation is favored below 90 °C. The experiment resulted in more than one optimal method for maximum yield of monomethylated compounds, while at the same time minimizing the other products. However, since the shortest possible reaction time at high temperatures and high pH values is desirable for anthocyanins, the following conditions were considered optimal: 95 °C for 90 min with 2.95 mmol of DBU. Under these conditions, a total yield of 8% monomethylated cyanidin-3-*O*-glucoside and 4% carboxymethylated cyanidin-3-*O*-glucoside was achieved. After the reaction, approximately 21% of cyanidin-3-*O*-glucoside remained unreacted. Approximately 40% of the compounds present after the reaction could be characterized (Table 1). Among the remaining 60%, the degradation products of cyanidin-3-*O*-glucoside, phloroglucine aldehyde, protocatechuic acid, and their methylated derivatives, were detected in the synthesis mixture. Whether these compounds are degradation products of methylated cyanidin-3-*O*-glucoside derivatives, or whether the methylation of these compounds took place after the degradation of cyanidin-3-*O*-glucoside, was not investigated.

### 2.3. Increase in Solvation of Anthocyanins

During the experiments, it was observed that although the solution took on a red color, some freeze-dried anthocyanin particles were still visible as a suspension in the DMC. This observation is also mentioned in the literature on the methylation of quercetin, where the yield after 72 h is only 30% [21]. To promote the conversion of anthocyanins, in addition to controlling the reaction by adjusting temperature, time, and catalyst concentration, measures to increase the solubility were considered. With this aim, experiments with the application of ultrasound and different solvents were used.

#### 2.3.1. Ultrasound

The overall influence of ultrasound on the synthesis was small. Compared to the synthesis under the optimized conditions, neither an improvement nor a deterioration was observed. It has been reported that the yield of the *O*-alkylation of 5-hydroxy-4-oxo-4H-chromene-2-carboxylic acid could be increased from 28% to 97% by ultrasound [23]. To achieve this, the authors performed 90-min continuous ultrasound treatment with an ultrasonic sonotrode at a frequency of 20 kHz while the system continued to be externally heated. Such an experimental setup could not be realized for the synthesis of methylated cyanidin-3-*O*-glucoside derivatives at the optimal temperature at 95 °C because the sonotrode (Hielscher Ultrasonics GmbH, UP200St) can be used only up to temperatures of approximately 70 °C. Furthermore, ultrasonic treatment during chemical synthesis might result in significant degradation of the products. The mechanisms associated with ultrasound are radical reactions [24]. Those types of reactions, against which anthocyanins may have a stabilizing effect due to their chemical properties, may then lead to countless isomeric polymeric compounds that are difficult to detect [18].

#### 2.3.2. Solvents

Another approach to obtain more initially dissolved cyanidin-3-*O*-glucoside molecules was the addition of different solvents. The synthesis followed the procedure mentioned under Section 3.2, whereby the sample substance was dissolved in 200 µL solvent such as methanol, DMF, dimethyl sulfoxide (DMSO), water, or pyridine before the addition of DMC and DBU and stirred vigorously. None of the selected solvents were able to increase the yield of monomethylated anthocyanins compared to a reaction without the addition of solvents. In fact, the yield actually decreased. The yield of the higher methylated cyanidin-3-*O*-glucoside derivatives did not exceed 2%. Although the solutions were perceived as more homogeneous before and after the reaction, the yield could not be improved. Although to our knowledge there are no reports of a reaction with the abovementioned solvents except water, where hydrolysis of DMC may occur, such reactions cannot be excluded. It is also possible that a solvent layer forms around the cyanidin-3-*O*-glucoside, and the DMC is unable to react with it. The loss of cyanidin-3-*O*-glucoside increased for all solvents: only between 2 and 4% of the initial amount could be detected without producing more methylated compounds.

A plausible explanation for this observation might be an improved heat transfer to the cyanidin-3-*O*-glucoside due to the increased solubility, and thus increased surface and contact possibility. When water is used as the solvent, it is possible that a water molecule reacts with the anthocyanin to form the colorless chromenol, which would no longer be detectable at 520 nm [8]. It should also be taken into account that nucleophilicity is strongly dependent on the solvent. Although DMSO and DMF should not have any influence on the nucleophilicity, as they are not able to form hydrogen bridge bonds, water can have an influence even in small quantities, as it can build hydrate shells around the cyanidin-3-*O*-glucoside hydroxyl groups.

The method presented may be further optimized using protective groups, as employed by Bouktaib et al., [5] or the use of phase transfer catalysts, as proposed by Tundo et al. [13].

### 2.4. Identification

Identification of the synthesized compounds was first carried out with ultra-violet (UV)/Vis detection at 520 nm, since anthocyanins have an absorption maximum in this region, and then by mass spectrometric analysis of the molecular and fragment ions using ultra-high-performance liquid chromatography coupled to electrospray ionization multistage mass spectrometry (UHPLC-ESI-LIT-MS^n^). Only those compounds that showed a loss of glucose ([M − 162]^+^) in the MS^2^ spectrum followed by the loss of a methyl group ([M − 162 − 15]^+^) in the MS^3^ spectrum were identified as methylated anthocyanins. To determine the accurate mass and collision cross section (CCS) values, LC-IMS-qTOF-MS was used. Table 1 gives a summary of the identification characteristics of cyanidin-3-*O*-glucoside and its derivatives at their respective degrees of methylation.

#### 2.4.1. Retention Times (RT)

**Figure 2:**
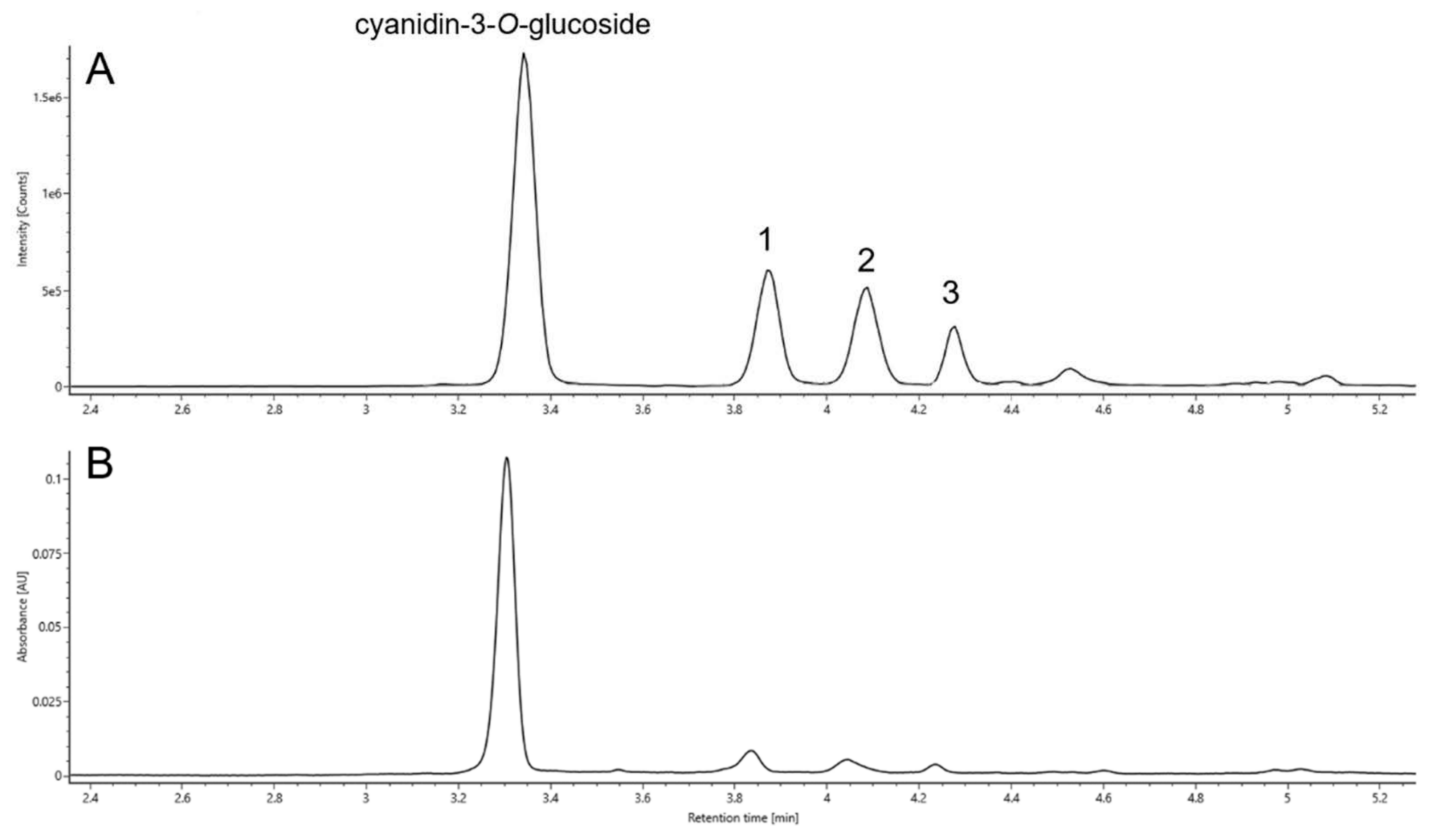
UHPLC-MS chromatograms of the reaction of cyanidin-3-*O*-glucoside with 2 mL DMC and 3.01 mmol DBU. (**A**), EIC of the *m*/*z* 449 ([M]^+^, cyanidin-3-*O*-glucoside) and the *m*/*z* 463 ([M]^+^, monomethylated cyanidin-3-*O*-glucoside, peaks **1–3**). (**B**), UHPLC-DAD chromatogram extracted at 520 nm.

Regarding the elution order in Figure 2, it is evident that methylated cyanidin-3-*O*-glucoside derivatives are, as expected, less polar. The higher the degree of methylation, the later the compounds are eluted. It can also be observed that the retention time of peak **2** is in accordance with that of the reference compound peonidin-3-*O*-glucoside (3′-OH methylated). Another methylated cyanidin-3-*O*-glucoside which may be used as reference is described in the literature as isopeonidin-3-*O*-glucoside (4′-OH methylated). It is formed as a metabolite of human and porcine enzymes [1,7]. Considering only those reports of authors who used acetonitrile (ACN) and water as eluents, the 3′-OH methylated compounds eluted earlier than the 4′-OH. Regarding the elution order of methylated quercetin or catechin derivatives, the literature indicates also that 3′-OH is eluted before 4′-OH, and 5-OH earlier than 7-OH [25,26,27]. Although the structure is different from the anthocyanins, it may be an orientation in elucidation.

#### 2.4.2. UV Spectra

Considering shifts in UV spectra after conjugation can provide hints about the position [28,29]. Unfortunately, the absorption maxima of the methylated derivatives are not significantly lower than that of cyanidin-3-*O*-glucoside. The absorption maxima of the monomethylated compounds and of cyanidin-3-*O*-glucoside are approximately 519 nm. Considering the ratio of λ_440 nm_ to λ_max_, differences become more noticeable. It can be seen that with 40% peak **2** and **3** have a different ratio than peak **1** with 33%, and the reference substances cyanidin-3-*O*-glucoside, peonidin-3-*O*-glucoside, and 7-*O*-methylcyanidin-3-*O*-galactoside have ratios of approximately 31%. The observations described in the literature, where methylation of cyanidin at the 3′-OH position almost doubles the ratio, could not be confirmed. However, the solvent used was methanol and not ACN [30]. Through the sulfation of cyanidin (unpublished results), the slightest change in this ratio was found at the B ring. If the UV shifts in the two reactions are similar, it can be assumed that peak **1** is derivatized at the B ring and peaks **2** and **3** at the A ring.

#### 2.4.3. Mass Spectrometry

**Figure 3:**
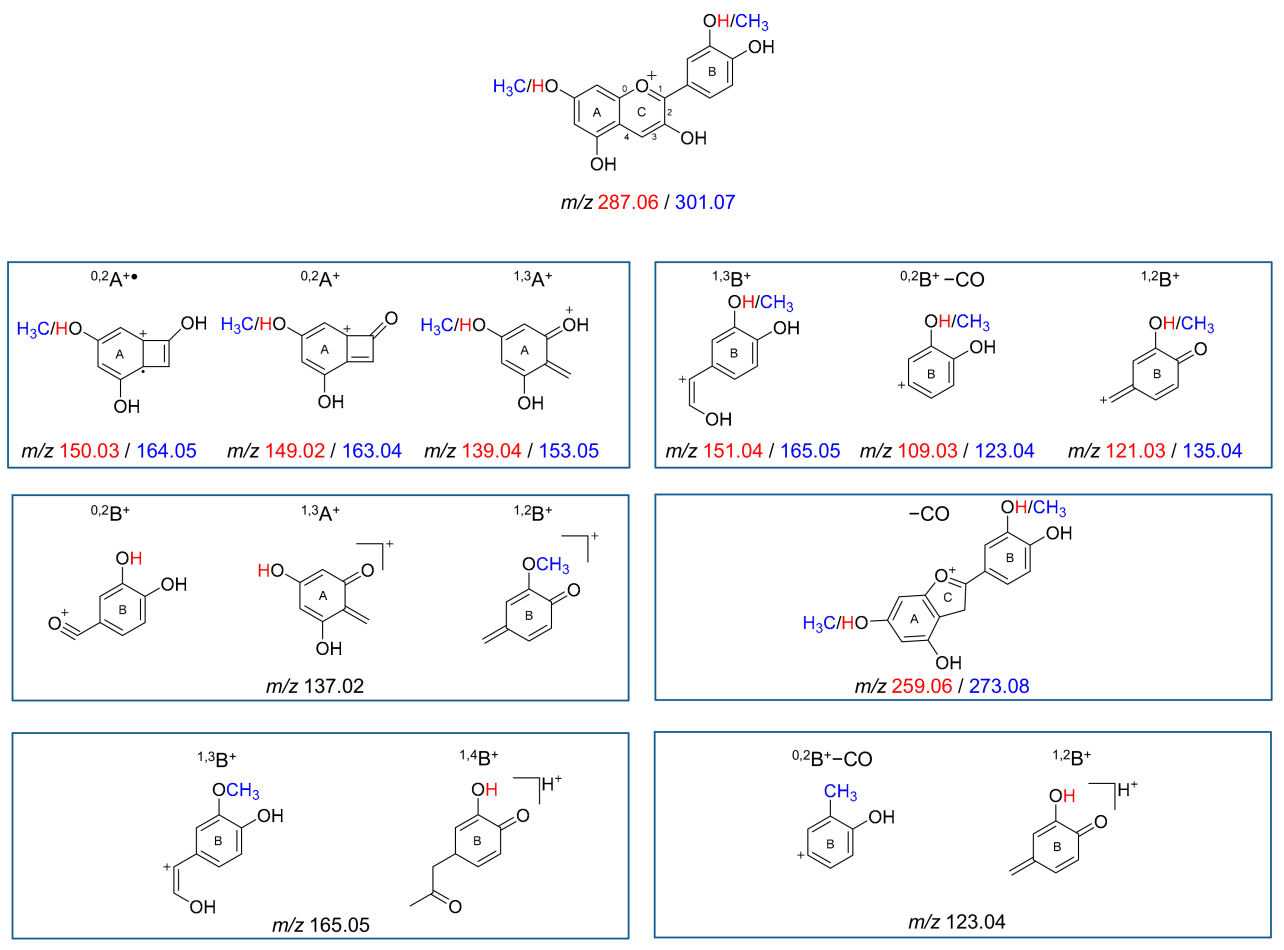
Postulated fragmentation scheme for cyanidin derivatives methylated on the A or B ring, based on the proposals of Barnes & Schug [32] and González-Manzano et al. [25].

As the MS^3^ spectra of the monomethylated derivatives (*m*/*z* 463; [M]^+^) represent the fragmentation behavior of the aglycone (*m*/*z* 301; [M − 162]^+^), these were used to determine their substitution pattern. The fragment at *m*/*z* 273 is characteristic of methylated cyanidin and marks the loss of CO of the aglycone ([M − 162 − 28]^+^). In the same way, the fragment at *m*/*z* 259 results from the additional loss of the methyl group (*m*/*z* 287; [M − 162 − 15 − 28]^+^). All these fragments can also be found in their homolytic cleavage product form [31]. Figure 3 shows fragments that can be identified as characteristic of the A ring (*m*/*z* 150, 149, 139) and the B ring (*m*/*z* 151, 121, 109) [11,32]. A simple consideration leads to the conclusion that depending on the substitution, characteristic fragments a mass increment of 14 u are detectable in the spectra of the monomethylated cyanidin-3-*O*-glucoside molecules. In the case of methylation of cyanidin-3-*O*-glucoside, however, this consideration cannot be applied. Since the methyl group is such a small fragment, some of the masses may also belong to other fragments. For example, the fragment at *m*/*z* 137 is described as a characteristic ^0,2^B^+^-fragment [32]. Nevertheless, this mass may also belong to a methylated fragment of the *m*/*z* of [^1,2^B]^+^ (*m*/*z* 123 + 14) or to the ^1,3^A^+^-fragment [25]. Other examples are the fragment at *m*/*z* 185 (cyanidin: [M − H_2_O − CO − CO − CO]^+^), which may also belong to a methylated fragment of cyanidin ([M + CH_3_ − H_2_O − CO − CO − C_2_H_2_O]^+^ (171 + 14)), the fragment at *m*/*z* 161 (cyanidin: [M − CO − CO − C_2_H_2_O − CO]^+^ or methylated cyanidin: [^1,4^B + CH_3_ − H_2_O]^+^), and the fragment at *m*/*z* 123 (cyanidin: [^1,2^B]^+^ or methylated cyanidin: [^0,2^B + CH_3_ − CO]^+^ (109 + 14)) [25,32].

#### 2.4.4. Ion Mobility Spectrometry

Over the drift time of the substances, CCS values can be calculated. These depend on the instrument settings, the *m*/*z*, and the structure and configuration of the molecule. Based on the retention time, it might be concluded that peak **2** is peonidin-3-*O*-glucoside. However, at 208.1 ± 0.3 Å^2^, the CCS value of peak **2** deviates too much from the CCS value of the reference with 207.0 ± 0.3 Å^2^. Thus, the possibility that the cyanidin derivative methylated at the 3′-OH position is among the three detected can be cautiously excluded.

#### 2.4.5. Views on Reactivity and Order of Methylation

As the ratios of the three monomethylated peaks to each other are 43:39:18, only 3 methylated derivatives were formed in significant quantities. Thus, the question remains regarding which OH group of cyanidin-3-*O*-glucoside is least reactive. In the case of quercetin, the 5-hydroxyl is by far the least acidic [25], whereas in cyanidin-3-*O*-glucoside, this position is the one with the highest pk_a_ after 7-OH [33]. The pk_a_ values for the OH groups in catechin are almost equal at approximately 9 [25]. Therefore, no regioselectivity in the methylation reaction of catechin is expected [25]. However, the lowest amount of catechin monomethylated is observed in position 5, with slightly more monomethylated in 7. Additionally, it is described that catechin is preferentially methylated at 3′ and 4′-OH [27], which may be due to a better accessibility.

In the past a methylation order that may be dependent on the acidity of the single OH groups of quercetin was observed, where the most acidic group is methylated first [34]. This might be used to identify the methylation position in anthocyanins, as their OH groups also show quite different acidities. According to this approach, the three most abundant resulting derivatives of cyanidin-3-*O*-glucoside are 7, 5, and 4′-OH. However, it should be noted that the conditions under which the work was carried out were completely different.

#### 2.4.6. Hydrolysis

**Figure 4:**
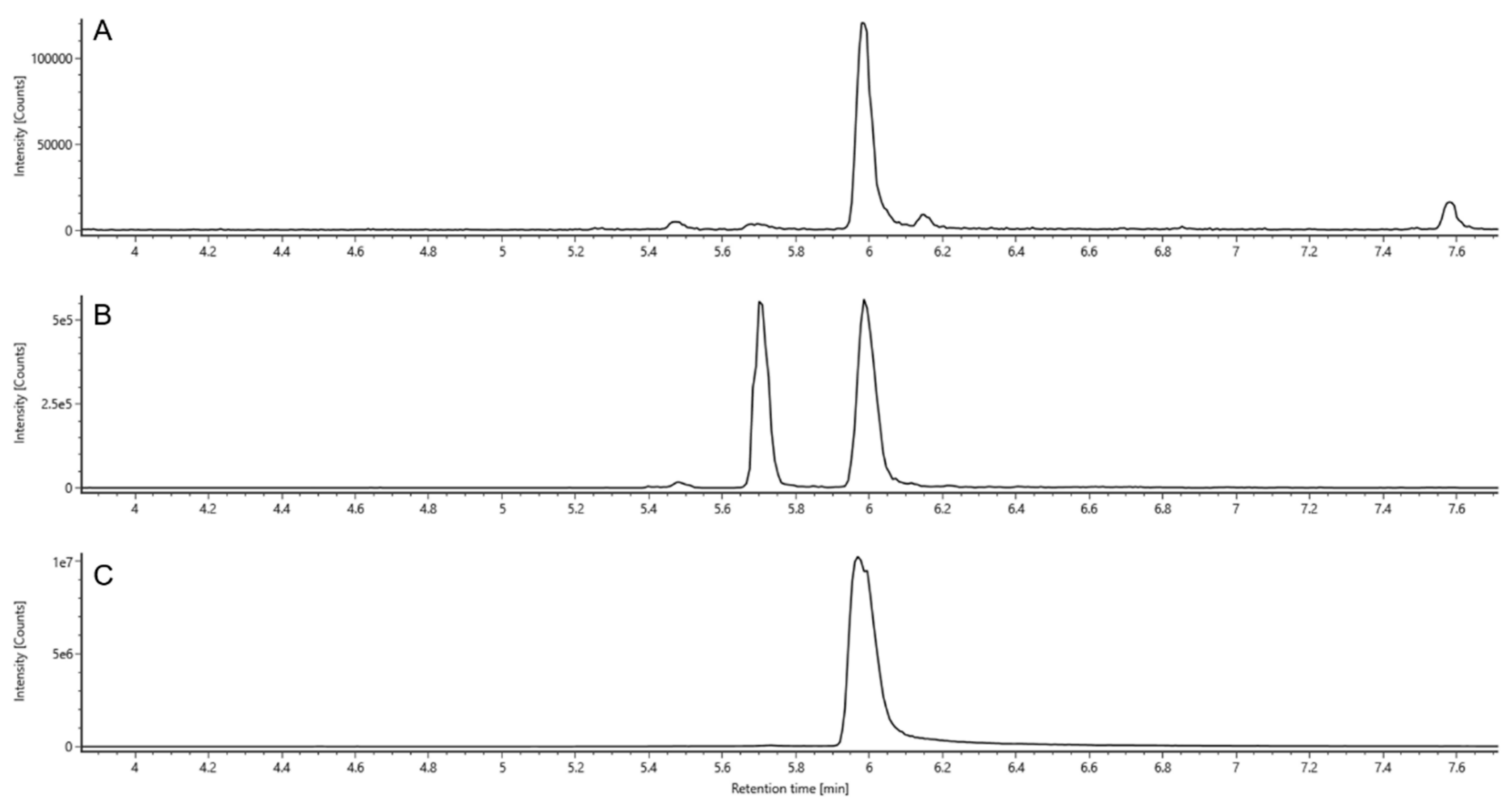
Extracted ion chromatogram of the *m*/*z* 301 ([M]^+^, methylated cyanidin) of the methylation of cyanidin-3-*O*-glucoside after hydrolysis (**B**) compared to a hydrolyzed sample of Brazilian pepper (**A**) and a peonidin reference (**C**).

To obtain more precise information about the aglycones, a sample was hydrolyzed with hydrochloric acid, as the hydrolysis allows the aglycone to be measured as MS^2^ rather than as MS^3^. The hydrolyzed sample was measured with both LC-ESI-LIT-MS^n^ and LC-IMS-qTOF-MS and compared with a peonidin standard and a hydrolyzed sample of Brazilian pepper extract. As Brazilian pepper contains 7-*O*-methylcyanidin galactoside, [28] a comparison of the glycosides was not sufficient for identification. The authors succeeded in distinguishing peonidin and 7-*O*-methylcyanidin based on characteristic fragments for the A ring and the B ring. Figure 4 shows an extracted ion chromatogram (EIC) of the [M]^+^, *m*/*z* 301 of a hydrolyzed cyanidin-3-*O*-glucoside sample after methylation compared to a hydrolyzed sample of Brazilian pepper and a peonidin reference.

Table 2 provides information about the aglycones found. In comparison with the reference substances, it can be seen from peak **18** that peonidin and 7-*O*-methylcyanidin have the same retention time. The observation that peonidin and 7-*O*-methylcyanidin have similar chromatographic behavior was already described before [30]. The two substances have almost the same Rf value in TLC. Fortunately, IMS is more accurate as an identification technique in this instance. The CCS values allow us to exclude that the detected peak **18** is peonidin because their values differ from each other by 160.3 ± 0.5 Å^2^ for peak **18** and 159.1 ± 0.1 Å^2^ for peonidin. However, the value of 7-*O*-methylcyanidin at 160.3 ± 0.5 Å^2^ is identical to that of peak **18**. Assuming that glucose does not change the elution order, it can be cautiously concluded that peak **3** is 7-*O*-methylcyanidin-3-*O*-glucoside.

Regarding the absorption maxima at approximately 520 nm, the tendency to undergo a hypsochromic shift as compared to cyanidin is more pronounced for the trimethylated derivatives and the tetramethylated derivative than for mono- and dimethylated cyanidin derivatives.

After hydrolysis, three monomethylated compounds can be found. Peak **16** is the smallest, and the ratio of the three is 1:45:54. An explanation for only two large peaks remaining after hydrolysis might be the coelution of two substances. On the other hand, one of the three compounds may have been decomposed by hydrolysis. This would mean that one of the methyl group positions might have a destabilizing effect on the cyanidin-3-*O*-glucoside molecule. It has been described that methylation in the B ring decreases stability in neutral media [35]. However, opposite observations under acidic conditions and high temperatures, showing a stabilizing effect of the methyl group of peonidin are also described [36]. Concerning the other OH groups, no report could be found.

## 3. Materials and Methods

### 3.1. Chemicals

LC-MS-grade water, acetonitrile, and formic acid were purchased from ChemSolute (Renningen, Germany). Methanol was obtained from VWR (Darmstadt, Germany). Dimethyl carbonate (DMC), dimethyl sulfoxide (DMSO), and 1,8-diazabicyclo[5.4.0]undec7.ene (DBU) were obtained from Carl Roth (Karlsruhe, Germany). *N,N*-dimethyl formamide (DMF) was purchased from Fisher Scientific (Waltham, Massachusetts). Peonidin and peonidin-3-*O*-glucoside chloride were obtained from Phytoplan (Heidelberg, Germany).

**Scheme 2:**
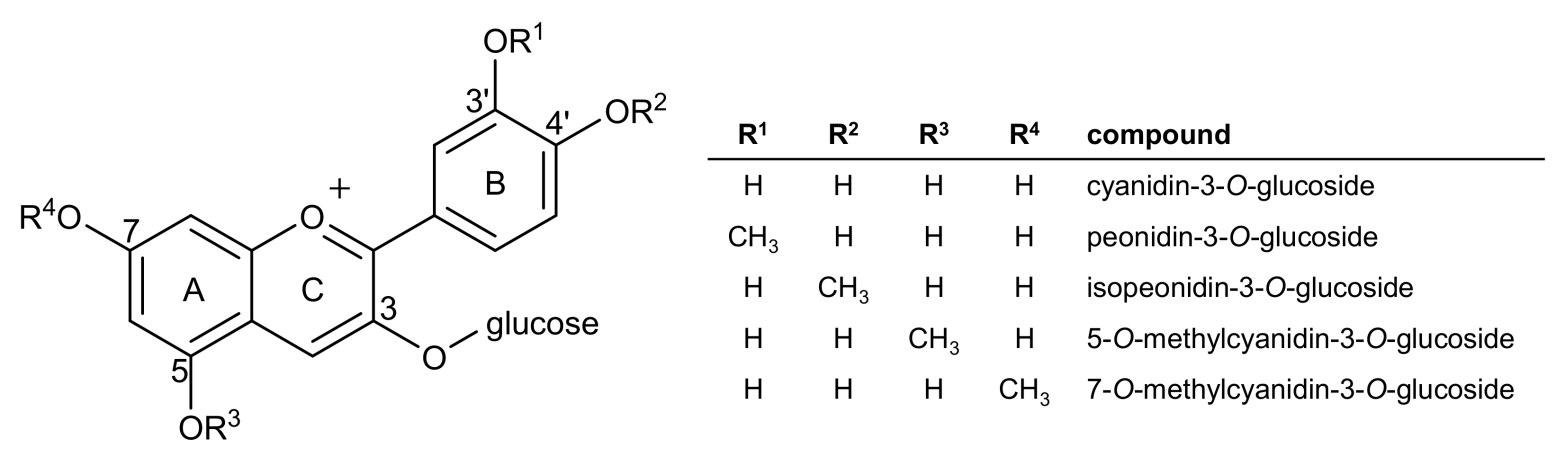
Structure of cyanidin-3-*O*-glucoside and its methylated derivatives.

An anthocyanin extract containing primarily cyanidin-3-*O*-glucoside (Scheme 2) was obtained as described before [9].

### 3.2. Hemisynthesis

Ten milligrams of the above-described anthocyanin extract containing approximately 20 µmol cyanidin-3-*O*-glucoside was reacted with 2 mL dimethyl carbonate, which served both as the reactant and the solvent. The reaction was catalyzed by 440 µL (2.95 mmol) DBU at 95 °C for 90 min. It was conducted in a 5 mL reaction vial with cap and septum in a heating unit under constant stirring. This approach was based on a method by Bernini et al. [21] and was optimized by a randomized, central composite design. The investigation was supported by the software Design Expert, which is suitable for response surface methods. A range of 60 °C to 100 °C was considered for the temperature, and a range of 30 to 480 min was used for the reaction time. The volumes of catalyst were chosen between 130 μL and 440 μL. The central point of the experimental design was thus 80 °C, 255 min, and 285 μL (1.91 mmol) DBU. This central point was repeated five times.

#### Sample Preparation

To the samples obtained through synthesis, 1 mL of methanol was added to remove excessive DMC azeotropes under nitrogen flow. The residue was dissolved in a mixture of acetonitrile, water, and formic acid (18.5/78.5/3; *v*/*v*/*v*) and adjusted to a volume of 5 mL. After further dilution (1:2 or 1:10) and microfiltration (regenerated cellulose, 0.2 µm), the samples were analyzed using liquid chromatography coupled with ion trap (LC-LIT-MS^n^) and ion mobility quadrupole time of flight (LC-IMS-QTOF-MS) multistage mass spectrometry.

For further identification purposes, the glucose moiety of the resulting reaction products was removed through hydrolysis with hydrochloric acid for 90 min at 90 °C. This procedure allowed the comparison with an anthocyanin-rich extract of Brazilian pepper (*Schinus terebinthifolius*), which was treated in the same way.

### 3.3. LC-MS Analysis

UHPLC analysis of the reaction products was performed on an Acquity UPLC I-Class system (Waters, Milford, MA, USA). The apparatus consisted of a sample manager cooled at 10 °C, a binary pump, a column oven, and a diode array detector scanning from 250 to 650 nm. The column oven temperature was set at 40 °C. An Acquity HSS-T3 RP18 column (150 mm × 2.1 mm; 1.8 µm particle size) was combined with a precolumn (Acquity UPLC HSS T3 VanGuard, 100 Å, 2.1 mm × 5 mm, 1.8 µm), both from Waters (Milford, MA, USA). The separation was performed with water (A) and acetonitrile (B) as eluents, both acidified with 3% (*v* + *v*) formic acid. The flow rate was set at 0.4 mL/min. Analyses of the methylated cyanidin-3-*O*-glucoside derivatives were carried out with linear gradient conditions from 7% B to 25% B for 5 min, then to 50% B for 5 min. For the methylated cyanidin derivatives, the linear gradient ranged from 7% B to 25% B for 5 min, then to 70% B for 9 min. The injection volume was 5 µL. For MS analysis, the UHPLC was coupled with an LTQ-XL ion trap mass spectrometer (Thermo Scientific, Inc., Waltham, MA, USA). This was equipped with an electrospray interface operating in positive ion mode. Ion mass spectra were recorded in the range of *m*/*z* 245–1000. The source voltage was kept at 4 kV at a current of 100 µA, and the tube lens was adjusted to 55 V. The capillary temperature was set at 325 °C with a spray voltage of 4 V. The sheath, auxiliary, and sweep gas was nitrogen at a flow of 70, 10, and 1 arb, respectively. Three consecutive scans were conducted: a full mass scan, an MS/MS scan of the most abundant ion from the first scan using a normalized collision energy (CE) of 65%, and an MS^3^ of the most abundant ion in MS^2^ with a CE of 65%. In addition, multiple reaction monitoring (MRM) measurements were performed on the fragment of the monomethylated aglycone (*m*/*z* at 301, [M]^+^) with different collision energies ranging from 10 to 200%. The Xcalibur (2.2SP1.48, Thermo Scientific, Inc., Waltham, MA, USA) was used to evaluate the data. For ion mobility spectrometry measurements, the UHPLC was connected to a Vion IMS QTOF mass spectrometer (Waters, MA, USA). The ionization mode was positive. The capillary voltage was 0.5 kV, the source temperature was 120 °C, the cone voltage was 40 V, the desolvation gas temperature was 550 °C, and the desolvation gas flow was 1200 L/h. Automatic lock correction was conducted every 5 min with leucine-enkephalin as the lock mass at a concentration of 100 pg/µL. The drift gas was nitrogen, and the MS mode was high definition with a low collision energy of 6 eV and a high collision energy ramp of 20–40 eV. Data were acquired and processed using UNIFI v1.9.2.045 (Waters, Milford, MA, USA).

## 4. Conclusions

With this work, a foundation for the green synthesis of methylated anthocyanin metabolites is provided. It was possible to maximize the synthesis of the desired monomethylated derivatives and minimize undesired side products. Cautious identification of 4′-, 5-, and 7-OH derivatives was possible. Precise identification after isolation would be the next step to use the synthesized components as reference substances. Nuclear magnetic resonance (NMR) spectroscopy is the most promising approach here, as other spectroscopic methods reach their limits. In addition, the methylation of other anthocyanins should be included to investigate whether the aglycone or the sugar moiety have an influence on the synthesis.

## Figures and Tables

**Table 1:** LC-DAD-ESI-IMS-qTOF results of the synthesis reaction of methylated cyanidin-3-*O*-glucoside.

Compound Number	[M]^+^ (*m*/*z*) Observed	[M]^+^ (*m*/*z*) Calculated	Mass Error (mDa)	RT (min)	CCS Value [M]^+^ (Å^2^)	Fragment Ions (*m*/*z*)	Yield (%) ^a^	λ_max_ (nm)
**Reference Substances**								
cyanidin-3-*O*-glucoside							21 ^b^	
	449.1078	449.1078	±0.0	3.42	202.8 ± 0.4	287.0551		518
peonidin-3-*O*-glucoside								
	463.1234	463.1235	−0.1	4.19	207.0 ± 0.3	286.0471, 301.0707		519
**Methylated Compounds**								
monomethylated cyanidin-3-*O*-glucoside							8	
1	463.1230	463.1235	−0.5	3.95	207.2 ± 0.2	286.0470, 301.0707		520
2	463.1228	463.1235	−0.7	4.16	208.1 ± 0.3	286.0473, 301.0704		517
3	463.1231	463.1235	−0.4	4.36	207.3 ± 0.3	286.0471, 301.0706		517
dimethylated cyanidin-3-*O*-glucoside							3	
6	477.1386	477.1391	−0.5	4.60	213.5 ± 0.4	300.0624, 315.0861		516
7	477.1397	477.1391	+0.6	4.99	212.9 ± 0.5	315.0862		518
8	477.1390	477.1391	−0.1	5.17	212.3 ± 0.1	300.0638, 315.0858		516
trimethylated cyanidin-3-*O*-glucoside							1	
9	491.1547	491.1548	−0.1	5.38	218.3 ± 0.2	329.1019		520
10	491.1543	491.1548	−0.5	5.97	216.5 ± 0.4	329.1026		518
11	491.1548	491.1548	±0.0	6.08	218.3 ± 0.3	329.1019		517
tetramethylated cyanidin-3-*O*-glucoside							1	
12	505.1686	505.1704	−1.8	6.97	223.3 ± 0.2	343.1201		518
carboxymethylated cyanidin-3-*O*-glucoside							4	
13	507.1132	507.1133	−0.1	4.45	215.7 ± 0.3	287.0544		515
14	507.1134	507.1133	+0.1	4.56	212.2 ± 0.3	287.0549		515
15	507.1134	507.1133	+0.1	5.00	212.8 ± 0.6	287.0551		521

^a^ expressed as peonidin-3-*O*-glucoside equivalents ^b^ remaining after reaction.

**Table 2:** LC-DAD-ESI-IMS-qTOF results of the synthesis reaction of methylated cyanidin-3-*O*-glucoside after acidic hydrolysis.

Compound Number	[M]^+^ (*m*/*z*) Observed	[M]^+^ (*m*/*z*) Calculated	Mass Error (mDa)	RT (min)	CCS Value [M]^+^ (Å^2^)	Fragment Ions MS (m/z)	λ_max_ (nm)	
**Reference Substances**								
cyanidin								
	287.0548	287.0550	−0.2	4.86	154.7 ± 0.2	213.0545, 231.06493, 241.04919, 269.04454	527	
peonidin								
	301.0711	301.0707	+0.4	5.98	159.1 ± 0.1	201.05474, 229.04958, 258.05195, 286.0471	527	
7-*O*-methylcyanidin								
	301.0702	301.0707	−0.5	5.99	160.3 ± 0.5	286.04672, 258.05171	524	
**Methylated Compounds**								
monomethylated cyanidin								
16	301.0706	301.0707	−0.1	5.48	160.6 ± 0.5	258.05262, 269.04454, 286.04732	n.d.	
17	301.0704	301.0707	−0.3	5.71	159.4 ± 0.2	258.05262, 286.04732	528	
18	301.0701	301.0707	−0.6	5.99	160.3 ± 0.5	201.05407, 213.05477, 229.04907, 258.05165, 286.04683	524	
dimethylated cyanidin								
19	315.0860	315.0863	−0.3	6.67	164.4 ± 0.3	272.06712, 300.06231	527	
20	315.0860	315.0863	−0.3	6.74	164.7 ± 0.6	257.04412, 201.05023, 272.06728, 301.06719,	528	
21	315.0858	315.0863	−0.5	6.97	164.9 ± 0.3	257.04467, 271.06016, 300.06297	523	
trimethylated cyanidin ^a^								
22	329.1011	329.1020	−0.9	7.78	170.0 ± 0.4	314.07906, 286.08402, 271.06265, 257.08148	526	
23	329.1017	329.1020	−0.3	7.94	170.0 ± 0.2	314.07861, 286.08251, 271.06018, 257.08116	524	
24	329.1014	329.1020	−0.6	8.09	170.9 ± 0.4	313.07039, 314.0769, 285.07596, 268.07533	524	
tetramethylated cyanidin								
25	343.1177	343.1176	+0.1	8.80	176.8 ± 0.2	327.08619, 312.06264, 299.09129, 285.07529	522	

^a^ one probably resulted from a carboxymethylated and trimethylated cyanidin-3-*O*-glucoside.

## Data Availability

Data is contained within the article.

## Sample Availability

Samples of the compounds are not available from the authors.
